# Motivation to Lead as Mediator of Relations Between the Dark Triad, Big Five, and Leadership Intention

**DOI:** 10.3389/fpsyg.2021.675347

**Published:** 2021-08-20

**Authors:** Jeffrey C. Kennedy, Kim Yin Chan, Moon-Ho Ringo Ho, Marilyn A. Uy, Oleksander S. Chernyshenko

**Affiliations:** ^1^Massey Business School, Massey University, Auckland, New Zealand; ^2^Nanyang Business School, Nanyang Technological University, Singapore, Singapore; ^3^School of Social Sciences, Nanyang Technological University, Singapore, Singapore

**Keywords:** leader emergence, Dark Triad, motivation to lead, leadership intention, Big Five

## Abstract

This study seeks to enhance the distal-proximal modeling of personality trait–leader emergence relationships by (1) distinguishing between the motivation to lead (i.e., the reasons why a person seeks leadership roles) and leadership intention (i.e., one’s expressed desire to claim a leadership role) and by (2) examining how the Dark Triad traits add to the Big Five personality factors in predicting three motivation to lead factors and leadership intentions. Using personality and careers aspiration data collected from 750 university students, we found that affective-identity and social-normative motivation to lead mediate the effects of distal traits on intentions. In contrast, non-calculative motivation to lead does not contribute to leadership intentions, which has important implications for organizations seeking selfless leaders. Narcissism explains variance in leadership intentions over and above that explained by extraversion; this contrasts with the studies of leader emergence, where the effect of narcissism disappears once extraversion is controlled. Overall, our findings validate the three-factor conceptualization of motivation to lead and illuminate the roles of both bright and dark personality factors in understanding individual desire to attain leadership roles.

## Introduction

Since the bivariate meta-analyses of Judge et al. (2002) linking the Big Five and leadership criteria, various theorists (e.g., Ng et al., 2008; Van Idekkinge et al., 2009; DeRue et al., 2011) have proposed distal-proximal models to explain how personality traits relate to leadership outcomes like emergence and effectiveness *via* various mediators. One important mediator is an individual’s self-reported motivation to lead (MTL). Luria and Berson (2013), for example, demonstrated that individuals with higher levels of MTL were more likely to engage in teamwork behaviors, which resulted in them being assessed as leader-like by their peers. In addition to this influence on leader emergence, MTL also predicted subsequent formal appointment into leader roles.

In their recent meta-analysis, Badura et al. (2020) confirmed the importance of MTL as a mediator in their Distal-Proximal Model of Motivation and Leadership. They also argued that MTL is best treated as three separate factors rather than as a general single factor. Unfortunately, there has been a tendency among researchers to treat MTL as a single factor representing one’s desire to seek leadership roles, either by using second-order factor modeling or by only using affective-identity MTL as a substitute for MTL (see section “Motivation to Lead Is Multidimensional”).

DeRue and Ashford (2010) describe the process of leader emergence as involving an identity construction process, whereby individuals claim leader roles and others grant such roles. In this paper, we suggest that leadership intentions precede the claiming action and are therefore a more proximal predictor of leader emergence (being accepted by others as a leader) than MTL factors (which concern the reasons why an individual may seek leadership roles). In this view, leadership outcomes only emerge after this motivation has transformed into an intention to lead – when motivational processes have given rise to volitional processes (Achtziger and Gollwitzer, 2018). In doing so, we refine Badura et al.’s distal-proximal modeling of personality traits, leader motivation, and leadership outcomes by distinguishing leadership intention as a more proximal construct to leader emergence than MTL.

As an outcome or dependent variable in the study of leadership that is often contrasted with effectiveness, leader emergence is typically operationalized either perceptually in terms of how a person is perceived by others to be leader-like (e.g., Judge et al., 2002) or behaviorally in terms of assuming leadership roles in leaderless group contexts (Ensari et al., 2011; see also Hanna et al., 2021 for more comprehensive discussion of variations in definition and operationalization of this construct). Some researchers have used self-reports of “leadership aspirations” to predict emergence using such criteria as career attainment (Schoon and Polek, 2011), occupational status (Schoon et al., 2007) and hierarchical advancement (Tharenou, 2001). Leadership aspirations have been operationalized in various ways from the single item of Singer (1989) “How much would you like to be in a leadership position?” to scales developed by Van Vianen and Keizer (1996) and Gray and O’Brien (2007). Recently, for example, Lechner et al. (2018) defined leadership aspirations as “the intention to become a leader in a business context,” but operationalized it using a measure of leadership motivation. In this paper, we build on the meta-analysis of Badura et al. (Badura et al., 2020; which showed that MTL is best conceptualized multidimensionally as a mediator between traits and emergence) to argue that leadership intentions are more proximal to leader emergence than MTL. Whereas MTL concerns the reasons for wanting to lead, leadership intention concerns the volition preceding “the actions people take to assert their identity as…a leader” (DeRue and Ashford, 2010, p. 631).

Research into links between personality and leadership has been broadened over recent years by the inclusion of “dark side” traits (such as the Dark Triad of narcissism, psychopathy, and Machiavellianism) in addition to “bright side” Big Five factors. Studies have demonstrated negative effects at both individual and organizational levels (e.g., Chatterjee and Hambrick, 2007; Volmer et al., 2016; Braun, 2017). The dark side traits of a leader may shape an organization’s culture, resulting in ongoing harm extending beyond the leader’s tenure (O’Reilly and Chatman, 2020). Narcissism has been linked to a desire for power (Macenczak et al., 2016), leader emergence (Brunell et al., 2008; Grijalva et al., 2015), and all three MTL factors (Badura et al., 2020). Beyond this, we have very little knowledge of how all three Dark Triad traits shape each of the three MTL factors and leadership intentions. Given the clear evidence of harm resulting from dark side leaders in positions of power, we need a better understanding of how Dark Triad traits relate to MTL factors in the formation of leadership intentions.

This paper focuses on refining Badura et al.’s model of leader emergence rather than effectiveness. We have two specific aims: (1) to distinguish between MTL (the reasons why a person seeks leadership roles) and leadership intention (i.e., one’s expressed desire to claim a leadership role) and (2) to examine how the Dark Triad of personality adds to the Big Five in predicting MTL facets and leadership intention. In doing so, we address the current gap in understanding regarding the role of Dark Triad traits in leader emergence while adding more rigor to distal-proximal models of leadership by showing the value of the multidimensional MTL construct in describing the “whys” or “motives” that explain individuals’ intentions to take up leadership roles, responsibilities, and training. By examining how the three MTL factors differentially mediate relationships between personality factors (Dark Triad and Big Five) and leadership intention, we reinforce the importance of viewing MTL as a multidimensional construct, and a mediator between personality traits and leadership intention – a better proxy (more proximal indicator) for leadership emergence.

### Motivation to Lead Is Multidimensional

Chan and Drasgow (2001) defined MTL as “an individual-differences construct that affects a leader’s or leader-to-be’s decisions to assume leadership training, roles, and responsibilities and that affect his or her intensity of effort at leading and persistence as a leader” (p. 482). They conceptualized MTL as a general, second-order factor with three factors: affective-identity (the extent one enjoys leading others and identifies as a leader), social-normative (the extent one treats leadership as a responsibility and duty), and non-calculative (the extent that one views leadership opportunities positively despite the potential costs and/or minimal personal benefits of leading). They showed empirically that the three MTL factors have different patterns of antecedents in both the Big Five personality traits and socio-cultural values.

Concerned that leader motivation had “not been more fully integrated into efforts aimed at understanding the nuanced nomological network of leadership processes” (p. 331; i.e., linking distal traits to leader emergence), Badura et al. (2020) highlighted the need to clarify the conceptualization and measurement of MTL. In a meta-analytic review based on 1,154 effect sizes from 100 primary studies, they confirmed different antecedents for the factors, as well as their low intercorrelations. Their meta-analysis presented path-analytic evidence that supporting MTL as an important mediator in their distal-proximal model linking traits with leader emergence and effectiveness.

Noting MTL’s importance for leadership emergence and effectiveness, they argued for the operationalization of MTL “as three separate motivational constructs instead of as one overarching construct” (p. 331). They noted that 40% of studies in their review, which used the Chan and Drasgow MTL measure used only a subset of the three factors. In some cases, affective-identity MTL is characterized as an intention to lead (e.g., Bergner et al., 2019), a usage which is inconsistent with the conceptualization of Chan and Drasgow (2001).

### The Need to Distinguish Leadership Intention From MTL in Distal-Proximal Models

Motivation and intentions are distinct constructs – Kanfer (1990) argued that motivational constructs “subsume the determinants and processes underlying the development of intentions” (p. 80). In building up their distal-proximal model of leader performance, Van Iddekinge et al. (2009) also made the point that motivational constructs are precursors of intentions to act. Research in entrepreneurship incorporates intentions as an important stage between traits and action (e.g., Zhao et al., 2010), there is no comparable construct in leadership research.

Intentions are important because they are the transition stage, whereby competing motivational tendencies are transformed into planning and action (Heckhausen and Heckhausen, 2018). Motivation to undertake a certain behavior does not automatically produce the behavior in the absence of volition, the active intention to pursue one goal over another (Achtziger and Gollwitzer, 2018).

In the distal-proximal model of leader emergence, MTL should precede the leader’s intention to lead. A person acting on such an intention is more likely to assert their identity as a leader (DeRue and Ashford, 2010) and to seek out opportunities to lead, to emerge as a leader in a group, or to pursue leadership training (Stiehl et al., 2015). There is a difference between wishful thinking (liking the idea of being a leader) and intention to act (i.e., translating a desire for leadership into specific actions). Distinguishing motivation from intention in the distal-proximal process therefore provides a more fine-grained framework for the analysis of trait-leadership emergence relationships.

Studies of leader emergence typically rely on perceptions of others, on assessments as to whether the observed person has characteristics consistent with being a leader (Judge et al., 2002), or, *via* the objective emergence of leaders in leaderless groups (e.g., Ensari et al., 2011). Much less attention has been given to individual self-nominations or intentional actions aimed at emergence into leadership roles. This contrasts with other fields, such as entrepreneurship research, where intentions are seen as an important stage linking distal predictors to emergence as an entrepreneur. Intentions represent a “conscious plan or decision to exert effort to enact the behavior” (Conner and Armitage, 1998, p. 1430) and have been shown to predict behavior in many applied fields (for a meta-analytic review, see Armitage and Conner, 2001). For these reasons, we include leader intentions as the final (most proximal) stage in our model.

### The Dark Triad and Leader Emergence

While the Big Five is the most common organizing framework for personality research, the Dark Triad is the most frequently studied cluster of dark personality features (Zeigler-Hill and Marcus, 2016). Paulhus and Williams (2002) introduced the Dark Triad label to describe three interrelated, overlapping yet distinct “offensive yet non-pathological” (p. 556) personality constructs – Machiavellianism, subclinical narcissism, and subclinical psychopathy. Paulhus (2014) suggested these dark traits may be facets of a second-order global factor for the dark side of personality on account of their inter-correlations. Acknowledging the overlap among the traits, Paulhus and Williams suggest that all three should be used when seeking to identify which has the strongest relationship with a given outcome, a position supported by Furnham et al. (2014).

Narcissism, Machiavellianism and, to a lesser extent, psychopathy have been studied in relation to leadership (see Furtner et al., 2017 for a recent review), but there is less known about the role of these traits in leader emergence. Narcissism is clearly associated with emergence (Grijalva et al., 2015) and MTL (Badura et al., 2020), and scholars have speculated that Machiavellianism (e.g., Judge et al., 2009) and psychopathy (e.g., Mathieu et al., 2015) may contribute to leader emergence. A recent meta-analysis by Landay et al. (2019) found a positive correlation between psychopathy and some indicators of leader emergence (e.g., number of leadership positions held) but not with other indicators (e.g., peer ratings of informal leadership).

In a literature review relating the Dark Triad to outcomes like leader effectiveness, managerial derailment, and abusive supervision, Spain et al. (2016) concluded that complex relationships exist between Dark Triad and leadership outcomes and called for careful attention to variables that may moderate or mediate such relationships. We thus focus our attention on understanding the relationship between the Dark Triad and the three MTL factors, and how these relate to leadership intention to deepen our understanding of leader emergence.

Of the three Dark Triad traits, narcissism is most intuitively associated with leader emergence. Narcissists are more likely to emerge as leaders in leaderless group discussions regardless of their individual performance on team tasks, and they tend to be given high ratings of leadership potential (Brunell et al., 2008; Nevicka et al., 2011). The link with leadership emergence was confirmed in the meta-analysis of Grijalva et al. (2015). Narcissists are also likely to seek leadership opportunities (Braun, 2017), suggesting a positive link to leader intentions.

Empirical relationships have also been established between the Dark Triad and MTL factors with the meta-analysis of Badura et al. (2020), reporting narcissism correlating positively with affective-identity MTL (*r*=0.51) and social-normative MTL (*r*=0.31) and negatively with non-calculative MTL (*r*=−0.17). Based on the above discussion, we hypothesize:

*H1a*: Narcissism correlates positively with leadership intention.

*H1b*: Narcissism correlates positively with affective-identity MTL.

*H1c*: Narcissism correlates positively with social-normative MTL.

*H1d*: Narcissism correlates negatively with non-calculative MTL.

We know of no studies reporting empirical relationships between psychopathy and Machiavellianism with the three MTL factors, which is a concern in view of speculations linking these dark traits with MTL factors. Citing Judge et al. (2009) and Furtner et al. (2017) wrote: “Machiavellian leaders are strongly manipulative and dishonest. They exhibit an extrinsic (calculative) form of MTL which reduce intrinsic work motivation of followers” (p. 87). This makes sense because Machiavellianism is intuitively associated with being calculative. We thus hypothesize:

H2: Machiavellianism correlates negatively with non-calculative MTL.

Landay et al. (2019) found a small but positive relationship between psychopathy and leader emergence (as indicated by rank, rate of promotion, or number of leadership positions held) in a meta-analysis of the limited number of empirical studies available. In contrast, there was a non-significant (weakly negative) relationship between peer ratings of leadership potential and psychopathy. Furtner et al. (2017) also suggested a specific link between psychopathy and low-social-normative MTL writing that they “exhibit a non-altruistic/antisocial MTL” (p. 92). We thus hypothesize:

H3a: Psychopathy correlates positively with leadership intention.

H3b: Psychopathy correlates negatively with social-normative MTL.

We do not state hypotheses regarding the possible direct effect of Machiavellianism on leader intentions. Some authors have speculated that personalized power motives may encourage those high on this trait to seek leadership positions, but there is no supporting empirical evidence. Furtner et al. (2017) suggest that psychopathic and Machiavellian leaders “are not interested in leadership *per se*” (p. 92) even though they may value some of the outcomes open to them from positions of leadership responsibility.

The conceptualization of MTL by Chan and Drasgow (2001) acknowledges its role in shaping decisions around the assumption of leadership responsibilities and roles. We therefore expect MTL to influence leader intentions, thus acting as a mediator between more distal Dark Triad personality traits and leader intentions. We thus hypothesize broadly:

H4: The relationship between Dark Triad traits and leader intentions is mediated by MTL factors.

## Method

### Participants and Procedures

Seven hundred sixty university students were recruited from a wide range of disciplines in a large comprehensive university in Singapore. All volunteered to participate in a follow-up survey conducted about 2months after an annual university-wide survey of students’ career motivations and intention. Both surveys were conducted with the Institutional Review Board approval and with informed consent. All participants were administered the follow-up survey online in a computer laboratory and compensated S$10. After screening the data, 10 cases were discarded as they failed our attention checks resulting in a final sample of 750 useable cases (45% males, 55% females; mean age=23.2years, SD=1.51years).

### Measures

#### MTL Factors

*MTL factors* were measured using the nine-item scale described in detail in Chan et al. (2012) comprising three-item subscales measuring affective-identity (e.g., “I am the kind of person who likes influencing and managing people more than doing anything else”), non-calculative (e.g., “I do not expect to get any privileges if I agree to lead or be responsible for a project”), and social-normative (e.g., “I feel that I have a duty to lead others if I am asked”) motivation. Confirmatory factor analyses showed that there was very little difference in the fit of a second-order factor model [with global and facet-level MTL; *χ*^2^=304.628, *df*=120, *p*<0.001, root-mean-square error of approximation (RMSEA)=0.045, comparative fit index (CFI)=0.94] from that of a first-order factor (or three factors only) model (*χ*^2^=340.06, *df*=128, p<0.001, RMSEA=0.047, CFI=0.93), which justified examining these constructs at both second and first-order factor levels. Reliability coefficients for global and facet-level MTL scales were above 0.7.

#### Leadership Intention

*Leadership intention* was also measured *via* the scale of Chan et al. (2012). Participants were asked for their agreement/disagreement on a five-point scale on three statements reflecting leadership intention (e.g., “I plan to become a general leader or manager in the near future”). Scale reliability was good at 0.74. Combining leadership intention items with MTL items in a single factor confirmatory factor analysis provided very poor model fit (*χ*^2^=906.267, *df*=54, p<0.001, RMSEA=0.145, CFI=0.66). A second-order factor model (with global and facet-level MTL) and separate leadership intentions factor had good fit (*χ*^2^=137.81, *df*=50, p<0.001, RMSEA=0.048, CFI=0.97), indicating appropriate discriminant validity between MTL and leadership intentions.

#### Big Five

*Big Five* factors were measured using 35 bipolar adjective markers from the scale of Goldberg (1992) that was administered in the follow-up survey. Participants were asked to indicate how each pair of adjectives described them on a 1–9-point scale (e.g., “silent-talkative” for extraversion, “disorganized-organized” for conscientiousness, “unkind-kind” for agreeableness, “angry-calm” for emotional stability, and “uninquisitive-curious” for openness to experience). Reliability coefficients for all seven-item Big Five scales were good, between 0.84 and 0.87.

#### Dark Triad

The 12-item “Dirty Dozen” measure of Jonason and Webster (Jonason and Webster, 2010; three four-item subscales) was also administered in the follow-up survey. Participants were asked how much they agreed (1=not at all, 5=very much) with statements like: “I have used deceit or lied to get my way” for Machiavellianism; “I tend to want others to admire me” for narcissism; “I tend to lack remorse” for psychopathy. Considering the short subscale lengths, reliability coefficients were acceptable for Machiavellianism (four-items) *α*=0.72 and narcissism (four-items) *α*=0.77. The reliability of the psychopathy subscale (four-items) *α*=0.60 was somewhat lower. However, positive inter-item correlations (averaging 0.27) and low to moderate correlations with the other two subscales support its retention. Due to model saturation, we could not compare the fit of a second-order factor model with that of the first-order factor only model. However, since the three DT scales were correlated from 0.21 to 0.51 and *α*=0.80 for global DT items, we examined the DT at both second- and first-order factor levels.

### Analysis

Our analysis began with a review of scale descriptives and intercorrelations enabling us to address H1–H3. Moving beyond bivariate correlations, we then used hierarchical regression to assess the ability of Dark Triad traits to account for variance in leader intentions, over and above that explained by the Big Five and MTL. Finally, H4 was tested using mediation analysis.

## Findings

### General-Factor and Facet-Level Dark Triad Relationships With MTL and Leadership Intention

Table 1 summarizes scale statistics and correlations in this study. At the global level, MTL correlates with leadership intention at *r*=0.51 but this pattern masks differences at the first-order factor level. Of the three MTL factors, only two (affective-identity and social-normative MTL) are correlated with leadership intention (*r*=0.59 and *r*=0.45 respectively). Similarly (as discussed below), the lack of correlation between global Dark Triad and MTL also masks important relationships at the factor level. These findings justify the treatment of MTL as three first order factors rather than as a global, second order factor.

**Table 1:** Scale descriptive statistics, reliabilities (in diagonals), and inter-scale correlations.

Variable		N items	Mean	SD	1	2	3	4	5	6	7	8	9	10	11	12	13	14	15	16
**Demographic variables**																				
1	Gender	n.a.	n.a.	n.a	n.a.															
2	Age	n.a.	23.2	1.5	−0.49^**^	n.a														
**Leadership variables**																				
3	Leadership Intention	3	11.9	2.1	−0.21^**^	0.12^**^	(0.74)													
4	Overall MTL	9	31.7	4.7	−0.16^**^	0.11^**^	0.51^**^	(0.75)												
5	AI-MTL	3	9.8	2.5	−0.21^**^	0.11^**^	0.59^**^	0.78^**^	(0.78)											
6	NC-MTL	3	10.1	2.3	−0.01	0.05	0.06	0.62^**^	0.11^**^	(0.73)										
7	SN-MTL	3	11.9	1.8	−0.11^**^	0.07	0.45^**^	0.75^**^	0.52^**^	0.20^**^	(0.71)									
**The Dark Triad**																				
8	Overall Dark Triad	12	33.3	6.4	−0.22^**^	0.00	0.19^**^	−0.01	0.25^**^	−0.35^**^	0.08^*^	(0.80)								
9	Machiavellianism	4	10.3	3.0	−0.16^**^	0.04	0.17^**^	0.04	0.27^**^	−0.26^**^	0.07^*^	0.85^**^	(0.72)							
10	Narcissism	4	13.4	2.9	−0.17^**^	−0.02	0.27^**^	0.04	0.24^**^	−0.31^**^	0.17^**^	0.73^**^	0.40^**^	(0.77)						
11	Psychopathy	4	9.6	2.4	−0.16^**^	−0.01	−0.02	−0.12^**^	0.03	−0.22^**^	−0.08^*^	0.71^**^	0.51^**^	0.21^**^	(0.60)					
**The Big Five**																				
12	Extraversion	7	41.2	9.5	−0.10^**^	0.06	0.43^**^	0.50^**^	0.60^**^	0.08^*^	0.37^**^	0.19^**^	0.22^**^	0.16^**^	0.03	(0.87)				
13	Agreeableness	7	48.3	7.0	0.05	0.01	0.14^**^	0.31^**^	0.17^**^	0.26^**^	0.25^**^	−0.22^**^	−0.20^**^	−0.03	−0.30^**^	0.38^**^	(0.84)			
14	Conscientiousness	7	48.9	7.9	0.04	0.02	0.23^**^	0.26^**^	0.22^**^	0.12^**^	0.24^**^	−0.19^**^	−0.15^**^	−0.07	−0.25^**^	0.23^**^	0.44^**^	(0.85)		
15	Emotional Stability	7	41.9	8.8	−0.05	0.04	0.15^**^	0.29^**^	0.20^**^	0.24^**^	0.16^**^	−0.20^**^	−0.13^**^	−0.23^**^	−0.10^**^	0.35^**^	0.40^**^	0.35^**^	(0.85)	
16	Openness to Experience	7	48.3	7.3	−0.12^**^	0.01	0.29^**^	0.38^**^	0.41^**^	0.09^*^	0.32^**^	0.12^**^	0.16^**^	0.11^**^	−0.02	0.49^**^	0.33^**^	0.34^**^	0.31^**^	(0.85)

*N*=750; MTL=motivation to lead; AI-MTL=affective-identity MTL; NC-MTL=non-calculative MTL; SN-MTL=social-normative MTL; and n.a. = not applicable.

* *p*<0.05;

** *p<* 0.01.

Global Dark Triad is significantly correlated with leadership intention (*r*=0.19). When we examine the three Dark Triad traits separately, narcissism has the strongest correlation with leadership intention (*r*=0.27; supporting *H1a*). We had insufficient grounds on which to state a hypothesis regarding Machiavellianism, but it also correlates significantly with leadership intention (*r*=0.17). The correlation with psychopathy is not significant, thereby rejecting H3a. Global Dark Triad is unrelated to MTL; but at the trait level, we note that psychopathy is weakly negatively correlated with global MTL (*r*=−0.12).

When considering the MTL factors, a more differentiated pattern emerges. Affective-identity MTL is positively correlated with narcissism (*r*=0.24, supporting *H1b*) and with Machiavellianism (*r*=0.27). Social-normative MTL correlates with narcissism (*r*=0.17, supporting *H1c*) but is uncorrelated with psychopathy (i.e., *H3b* is not supported). Non-calculative MTL correlates negatively with all three Dark Triad traits – narcissism (*r*=−0.31 supporting *H1d*), Machiavellianism (*r*=−0.26 supporting *H2*), and psychopathy (*r*=−0.22).

It is thus meaningful to study the relationships between the Dark Triad and MTL constructs at the lower-order factor-level because all three Dark Triad traits have different relationships with MTL factors. Interestingly, low non-calculative MTL (reflecting calculativeness or a lack of motivation to make personal sacrifices when leading) appears to be a common MTL factor that relates to all three Dark Triad traits.

### Hierarchical Modeling to Examine Incremental Validity of Dark Triad Over Big Five

Table 2 summarizes various hierarchical regression models examining the incremental validity of Dark Triad over Big Five in predicting leadership intention. Model A includes age and gender as control variables. Controlling for gender and age, we observe from Models B1 and B2 that significant amounts of variance in leadership intention are accounted for by the Big Five (*R*^2^=0.24) and the Dark Triad (*R*^2^=0.12). Models B1, B2, and C reveal that the Dark Triad adds incremental validity to predicting leadership intention with extraversion (*β*=0.33), narcissism (*β*=0.20), and conscientiousness (*β*=0.17) as significant predictors.

**Table 2:** Hierarchical regression of Leadership Intention on the Dark Triad, Big Five factors, and respective motivation factors.

Dependent variable	Leadership intention													
Model	A		B1		B2		C		D1		D2		E	
Gender	−0.19	^**^	−0.16	^**^	−0.17	^**^	−0.13	^**^	−0.09	^**^	−0.08	^*^	−0.08	^*^
Age	0.02		0.04		0.01		0.03		0.01		0.02		0.02	
Machiavallianism			0.13	^**^			0.04				−0.01		−0.01	
Subclinical Narcissism			0.22	^**^			0.20	^**^			0.13	^**^	0.14	^**^
Subclinical Psychopathy			−0.16	^**^			−0.09	^*^			−0.05		−0.04	
Affective Motivation									0.47	^**^	0.45	^**^	0.38	^**^
(Non-)Calculative Motivation									−0.04		0.00		0.00	
Social-normative Motivation									0.20	^**^	0.18	^**^	0.16	^**^
Extraversion					0.38	^**^	0.33	^**^					0.11	^**^
Agreeableness					−0.08	^*^	−0.10	^*^					−0.07	
Conscientiousness					0.18	^**^	0.17	^**^					0.11	^**^
Emotional Stability					−0.04		0.03						0.03	
Openness to Experience					0.06		0.04						−0.02	
*R* ^2^	0.05	^**^	0.12	^**^	0.24	^**^	0.28	^**^	0.38	^**^	0.40	^**^	0.41	^**^
Adj *R*^2^	0.05		0.11		0.23		0.27		0.38		0.40		0.40	

* *p*<0.05;

** *p<* 0.01.

Model E adds the three MTL factors to the model. Narcissism continues to provide significant incremental validity (*β*=0.14) in explaining variance in leadership intention beyond the Big Five and MTL factors. Comparing models C (Dark Triad and Big Five) and E (with the addition of MTL), the MTL factors contribute a significant increment in variance accounted for in leadership intentions (*DR*^2^=0.41–0.28=0.13). Taken together, models D1, D2, and E indicate that affective-identity and social-normative (but not non-calculative) MTL explain significant amounts of variance in leadership intention even when Big Five and Dark Triad traits are included as predictors. This supports treating MTL and leadership intention as separate concepts while also reinforcing the call of Badura et al. (2020) to operationalize MTL factors as three separate constructs.

### Direct and Indirect Effects of Personality on Leadership Intentions Mediated by MTL

Mediation was analyzed with Mplus using maximum likelihood estimation with robust standard errors (MLR). Gender and age (controls) along with Big Five and Dark Triad traits were entered as independent variables. The three MTL factors were included as mediators, with leadership intentions as the dependent variable. The model also allowed for direct effects of controls and personality traits on leadership intentions.

**Figure 1:**
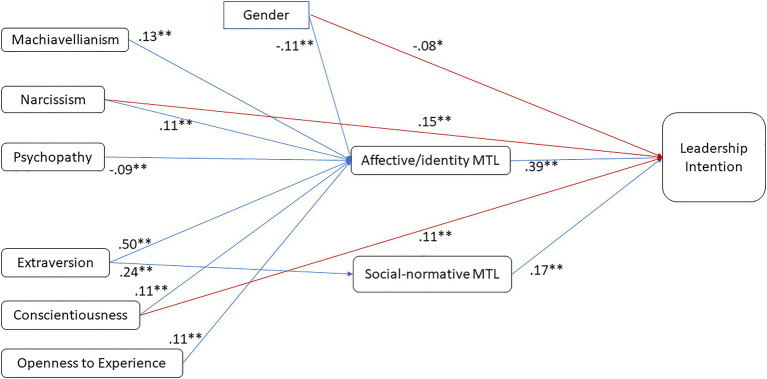
Mediation model showing significant direct and indirect paths. *p<0.05; **p<0.001. Only direct paths or paths that contribute to significant indirect effects (at 0.01 level) are shown.

Table 3 summarizes the results of the mediation analysis, showing values for (and significance of) direct and indirect paths. The indirect path coefficients shown in Table 3 are the product of the two paths X→M (e.g., Extraversion→affective-identity MTL) and M→Y (e.g., affective-identity MTL→leadership intentions). Figure 1 shows the separate X→M and M→Y path coefficients for all significant indirect paths (*p*<0.01).

**Table 3:** Standardized Indirect and Direct Effects of Personality Traits on Leadership Intentions.

	Indirect effects of personality traits on leadership intention *via* MTL factors									Direct effect		
	Affective-Identity			Non-Calculative			Social-Normative			Leadership Intention		
	Coeff.	*SE*	*p*	Coeff.	*SE*	*p*	Coeff.	*SE*	*p*	Coeff.	*SE*	*p*
Extraversion	0.192	0.026	<0.000	0.000	0.001	0.901	0.040	0.012	0.001	0.108	0.046	0.019
Agreeableness	−0.040	0.016	0.013	0.001	0.006	0.899	0.009	0.008	0.239	−0.069	0.040	0.084
Conscientiousness	0.043	0.015	0.005	0.000	0.002	0.900	0.019	0.008	0.021	0.114	0.038	0.002
Emotional Stability	0.008	0.015	0.608	0.000	0.003	0.899	−0.001	0.006	0.848	0.028	0.038	0.472
Openness	0.041	0.015	0.005	0.000	0.001	0.900	0.020	0.010	0.039	−0.018	0.040	0.657
Machiavellianism	0.050	0.016	0.002	−0.001	0.004	0.900	0.002	0.007	0.762	−0.011	0.036	0.765
Narcissism	0.040	0.015	0.009	−0.001	0.008	0.899	0.021	0.009	0.015	0.146	0.040	<0.001
Psychopathy	−0.036	0.014	0.010	0.000	0.003	0.900	−0.014	0.008	0.073	−0.045	0.035	0.201
Age	0.007	0.013	0.590	0.000	0.000	0.920	0.004	0.007	0.510	0.020	0.034	0.560
Gender	−0.041	0.013	0.001	0.000	0.003	0.900	−0.009	0.007	0.194	−0.084	0.036	0.021

*R*^2^ values for observed variables: Affective-Identity MTL=0.436 (*p*<0.001); Non-Calculative MTL=0.190 (*p*<0.001); Social-Normative MTL=0.206 (*p*<0.001); and Leadership Intention=0.395 (*p*<0.001). Age and gender were included as control variables; for correlations with gender, negative correlations indicate that men scored higher than women.

This analysis indicates the extent to which the effect of distal personality traits on leadership intentions is mediated by MTL factors. As non-calculative MTL was unrelated to intentions, the following discussion focuses on the mediation *via* affective-identity and social-normative MTL.

Considering Big Five traits first, affective-identity MTL mediated the effects of three traits on leadership intentions – extraversion (fully mediated, indirect path estimate=0.192), conscientiousness (partially mediated, 0.043), and openness (fully mediated, 0.041). In addition to its indirect effect *via* affective-identity MTL, conscientiousness also had a direct effect on leadership intentions (0.114). Only extraversion affected intentions *via* social-normative MTL (0.040), and its effect was fully mediated.

With respect to the Dark Triad, all three traits showed indirect effects on leadership intentions *via* affective-identity MTL – Machiavellianism (fully mediated, indirect path estimate=0.050), narcissism (partially mediated, 0.040), and psychopathy (fully mediated, −0.036). None of the paths *via* social-normative MTL reached significance at *p*<0.01. However, narcissism also had a direct effect on leadership intentions (0.146).

These findings partially support H4. With Big Five traits included in the model, affective-identity MTL mediated the effect of Dark Triad traits on leader intentions. As narcissism also had a direct effect, the mediation for this trait is partial.

## Discussion

Understanding relationships between the Dark Triad and leader emergence requires careful disentangling of the influence of variables, such as motivations and intentions, and attention to the level of measurement of both Dark Triad and MTL constructs. The global Dark Triad correlates significantly with leadership intentions but not with global MTL. It is only at the individual Dark Triad trait level that links between these dark-side personality traits and leadership motivation become clear.

Higher levels of narcissism and Machiavellianism are associated with higher levels of affective-identity MTL, suggesting an enjoyment of leadership roles and a degree of self-identification as leaders. However, these traits also correlate negatively with non-calculative MTL. This implies that increased levels of narcissism and Machiavellianism underlie a more calculative motivation for taking on leadership roles – a desire to benefit personally and a reduced willingness to make personal sacrifices in the fulfillment of leadership responsibilities. Thus, while Narcissism and Machiavellianism predict leadership intentions, those intentions appear to have a strong self-serving element. Higher levels of psychopathy are also associated with a reduced willingness to accept the costs of leadership but seem to have no bearing on leadership intentions.

These findings further validate the three-factor conceptualization of MTL by Chan and Drasgow (2001) and reinforce the call of Badura et al. (2020) for further research to understand the three MTL factors. By exploring the role of personality (both Dark Triad and Big Five traits) in shaping MTL and leadership intention, we also respond to the call of Judge et al. (2009) for more empirical research on the Dark Triad alongside the “brighter” Big Five personality factors with leader emergence.

Our findings regarding the relationship between narcissism and leadership intentions are particularly interesting. Grijalva et al. (2015) found that the positive effect of narcissism on leader emergence became non-significant when extraversion was included in their meta-analytic regression analysis. They concluded that while “narcissistic individuals were more likely to become leaders…this positive relationship was completely explained by the overlap between narcissism and extraversion” (p. 27). While this may make sense in the case of leader emergence based on peer observations of visible behavioral cues to assess leadership potential, our mediation analysis shows that links with leadership intentions are more complex. With all Big Five traits included, narcissism explained additional variance in leadership intentions both directly and indirectly (*via* affective-identity MTL). There is something about narcissism, which contributes to active intentions to pursue leadership opportunities over and above the effect of extraversion. This finding highlights the value of considering intentions as a proximal predictor of leader emergence in distal-proximal models of leadership.

Our finding that leadership intentions are related to affective-identity and social-normative MTL, but not to non-calculative MTL, provides a more nuanced understanding of the role of MTL in becoming a leader. Non-calculative motivation is based on a recognition of the potential costs of leading, and individuals high on this motivation can be viewed as selfless (or even reluctant) leaders. From this perspective, the lack of correlation between non-calculative MTL and leadership intentions is unsurprising. In contrast, Badura et al. (2020) found that non-calculative MTL was positively related to leadership emergence. This is consistent with the point of DeRue and Ashford (2010) that individuals may be endorsed as leaders in a social context even if they do not perceive themselves as possessing relevant leader attributes. Emergence and intentions are not the same, and it is important to maintain the distinction.

Developing a deeper understanding of non-calculative MTL is likely to be important in contexts, where organizations or society rely on people being willing to take on leadership roles despite costs incurred. Such costs could be financial or come in the form of reduced work-life balance, loss of privacy, reputational risk, and the like. Our finding (that people willing to accept these costs are not necessarily going to put their hand up for leadership roles) highlights the need for active recruitment and development of non-calculative leaders.

Our findings regarding non-calculative MTL also suggest value in future research exploring the emergence of servant (Van Dierendonck, 2011) or even self-sacrificial leadership (De Cremer et al., 2009). Maurer et al. (2017) have shown how an error management culture in an organization can contribute to employees leading out of a sense of duty and responsibility (social-normative MTL). What are the ways in which organizations (and organization cultures) vary in the extent to which they encourage or support the transition of employees high on non-calculative MTL into leadership roles?

Our mediation results are consistent with the finding of Badura et al. (2020) that only two MTL factors (affective-identity and social-normative) act as mediators between distal traits and leadership outcomes. According to Badura et al. leadership motives originating from pure enjoyment (affective-identity MTL) and being prompted to lead out of obligation (social-normative MTL) partially explained links between Big Five traits and leader emergence; however, there was no evidence that being motivated to lead out of selflessness (non-calculative MTL) promoted leader emergence. Our study extends this finding to Dark Triad traits.

While narcissism has received considerable research attention, there is a paucity of research into the role of Machiavellianism and psychopathy in leadership emergence or intentions. We confirmed the positive relationship of narcissism to leadership intentions and provide the initial evidence that Machiavellianism also contributes positively to intentions to lead. We found no relationship between leader intentions and psychopathy, which contrasts with the finding of Landay et al. (2019) regarding leader emergence. This may reflect the difference between intentions and the measures of emergence used in the studies analyzed by Landay et al., or just be further evidence that the relationship between psychopathy and becoming a leader is relatively weak.

### Research and Practical Implications

Future research could examine the processes by which personality traits influence MTL factors. For example, Guillén et al. (2015) demonstrate that self-comparisons by a person in respect to leadership models influence that individual’s leader self-efficacy and MTL. To what extent do personality traits (e.g., narcissism vs. conscientiousness) influence the choice of leader exemplars, and how do these choices subsequently influence MTL factors and leader emergence?

Similarly, Schyns et al. (2020) have found that congruence between implicit self and leadership theories influence affective MTL. Interestingly, congruence with respect to the negative component of self and leadership theories included in the study (manipulation) had no effect on MTL; they speculated that this may have resulted from roughly equal numbers of participants viewing this as positive vs. negative for leadership. Dark Triad trait levels are likely to influence the extent to which a potential leader views such behaviors as consistent with effective leadership and to thus influence their intentions regarding assuming leadership roles in different contexts.

Our study highlights the importance of distinguishing between the contribution of MTL and leadership intentions to leader emergence. Organizations require talented people who are willing to take on leadership roles and are capable of performing them effectively. While MTL represents an interest in becoming a leader, it requires intention for this desire to be translated into action. All Dark Triad traits had significant negative correlations with non-calculative MTL, but this factor of MTL had no correlation with intentions. Thus, the negative relationship with Dark Triad traits did not act to reduce leadership intentions. Those people motivated to accept the costs of leadership, to take on responsibility without seeking personal benefit, are no more likely to actively seek leadership roles than those low on non-calculative MTL. We therefore encourage organizations to take active steps to encourage and support non-calculative leaders to consider leadership roles, rather than rely on them to be proactive.

Our findings raise important practical considerations regarding the development and promotion of leaders. Given that increased levels of narcissism and Machiavellianism contribute to higher MTL and leadership intentions, organizations who wish to avoid promoting such people into leadership roles will need to have effective screening processes. These could include psychometric assessments of Dark Triad traits, together with sufficient observer data (e.g., from upward or peer assessments) to identify the dysfunctional narcissistic or Machiavellian behaviors. Grijalva et al. (2015) note that narcissism increases the likelihood that a person will be seen as leader-like, especially among people who have spent limited time together. As they point out, “the ugly side of narcissism takes time to emerge” (p. 28), suggesting that firms need to gather more comprehensive information on candidates than is provided by interviews or a typical assessment center.

### Limitations

Some limitations should be considered when interpreting the findings. This study is based on cross-sectional self-report data from a large sample of university students with an abbreviated nine-item MTL scale. Future research should use longitudinal designs to test our mediation model. Use of short scales like the Dirty Dozen to measure the Dark Triad is recognized to thread a “fine line” between construct accuracy vs. efficiency (Jonason and Luévano, 2013), but likely contributed to the relatively low reliability of our psychopathy measure. The 27-item MTL measure of Chan and Drasgow (2001) and longer measures of the Dark Triad (e.g., Hogan and Hogan, 1997) should also be used in future studies where possible. While we have tried to mitigate the possible effect of common method variance (e.g., by measuring personality 2months after measuring MTL and intentions), future research should obtain objective indicators beyond self-reports where available. The generalizability of our findings should be further verified with samples from other cultural and employment settings.

Finally, this paper focuses on understanding the role of the Dark Triad and MTL in the leader emergence process (Acton et al., 2019). We do not address leader effectiveness, but hope that our findings are timely when considered alongside the recent empirical evidence of Auvinen et al. (2020) showing how leader motivation profiles (conceptualized *via* the three MTL factors) relate to important outcomes at work (e.g., quality of leader-member exchange and well-being at work) and in recent attempts to understand leadership development in terms of the linkages between emergence and effectiveness (cf. Luria et al., 2019).

## Data Availability Statement

The raw data supporting the conclusions of this article will be made available by the authors, without undue reservation.

## Ethics Statement

The studies involving human participants were reviewed and approved by the Institutional Review Board, Nanyang Technological University. The patients/participants provided their written informed consent to participate in this study.

## Author Contributions

KC, M-HH, MU, and OC contributed to the design of the study. JK and KC wrote the manuscript. M-HH carried out data analysis. All authors contributed to manuscript revision, read, and approved the submitted version.

## Conflict of Interest

The authors declare that the research was conducted in the absence of any commercial or financial relationships that could be construed as a potential conflict of interest.

## Publisher’s Note

All claims expressed in this article are solely those of the authors and do not necessarily represent those of their affiliated organizations, or those of the publisher, the editors and the reviewers. Any product that may be evaluated in this article, or claim that may be made by its manufacturer, is not guaranteed or endorsed by the publisher.
